# Density measurement of samples under high pressure using synchrotron microtomography and diamond anvil cell techniques

**DOI:** 10.1107/S0909049510008502

**Published:** 2010-04-01

**Authors:** Xianghui Xiao, Haozhe Liu, Luhong Wang, Francesco De Carlo

**Affiliations:** a XOR, Advanced Photon Source, Argonne National Laboratory, Argonne, IL 60439, USA; b Natural Science Research Center, Academy of Fundamental and Interdisciplinary Sciences, Harbin Institute of Technology, Harbin 150080, People’s Republic of China

**Keywords:** high pressure, equation of state, microtomography, incomplete data

## Abstract

Accurate mass density information is critical in high-pressure studies of materials. It is, however, very difficult to measure the mass densities of amorphous materials under high pressure with a diamond anvil cell (DAC). Employing tomography to measure mass density of amorphous samples under high pressure in a DAC has recently been reported. In reality, the tomography data of a sample in a DAC suffers from not only noise but also from the missing angle problem owing to the geometry of the DAC. An algorithm that can suppress noise and overcome the missing angle problem has been developed to obtain accurate mass density information from such ill-posed data. The validity of the proposed methods was supported with simulations.

## Abstract 2

An algorithm is developed to extract accurate mass density information from tomography data of a sample embedded in a diamond anvil cell in a high-pressure environment.

## 1. Introduction

Research on the equation of state of materials under high-pressure conditions provides important information on the fundamental physical properties (*e.g.* density *versus* pressure) of materials, and is a traditionally active area in high-pressure research. For crystalline samples the routine method used to study density under high pressure is X-ray diffraction. However, for non-crystalline samples, such as amorphous materials and melts, it is challenging to obtain density information under high-pressure conditions. Using X-ray scattering and diamond anvil cell (DAC) methods, several cases have been reported by fitting the structural factors of the non-crystalline samples under pressure conditions to estimate the density (Eggert *et al.*, 2002 ▶; Shen *et al.*, 2002 ▶, 2004 ▶). More attention was recently attracted by combining scattering data with first-principle calculations and Monte Carlo simulations (Sheng *et al.*, 2006 ▶, 2007 ▶; Zaug *et al.*, 2008 ▶). At the same time, synchrotron X-ray absorption methods are widely used by applying the absorption law [*I* = *I*_0_exp(−μρ*t*), where *I* and *I*_0_ are the intensities of the transmitted and incident beams, respectively, μ and ρ are the mass absorption coefficient and density of the sample, and *t* is the sample length in the X-ray path] to measure the density of melts under pressure using a large-volume press (Katayama *et al.*, 1993 ▶, 1998 ▶; Katayama, 1996 ▶; Sanloup *et al.*, 2000 ▶). In these experiments the sample length measurement, which was normally based on a one-dimensional scan, was critical to accurately obtain sample mass density. To improve this method, synchrotron radiography techniques were developed to measure the density of melts under pressure (Chen *et al.*, 2000 ▶, 2005 ▶). Approaches to combining the synchrotron X-ray diffraction and absorption techniques for the density measurement of melts and amorphous materials in a DAC were also reported (Shen *et al.*, 2002 ▶; Hong *et al.*, 2007 ▶).

Recently, a tomographic method using a modified Drickamer anvil apparatus was introduced to study the density of melts (Wang *et al.*, 2005 ▶), and the authors discussed the possibility of applying tomography techniques to a DAC for higher pressure by pointing out that the reduction in sample volume in a DAC may limit the usefulness of this technique. In this report we present a methodology which is developed to overcome this problem for density measurements using microtomography in a DAC. In this work methods are proposed that aim to extract mass density information from both the complete data case and the case of data with missing angle, based on simulation results. In the complete data case, the mass density calculated from the tomographic reconstruction can be used directly to determine the compression curve of a material. In the case of data with missing angle, the relative mass density change of an unknown sample can be obtained with a known sample as reference. The methods may have wide applications for other important cases with amorphous materials and melts under much higher pressure conditions in materials science and Earth science.

## 2. An algorithm to extract mass density from tomographic reconstruction of a sample in a DAC

Tomography is an imaging technique that can build a three-dimensional (3-D) structure of a specimen from a series of images taken at many angles between 0 and 360° (Natterer, 1986 ▶; Kak & Slaney, 1987 ▶). Depending on the structure information encoded in the images, 3-D structures of elemental distribution, chemical state, phase *etc.* can be obtained. When the images are absorption-contrast-based projection images, tomography gives a 3-D map of the linear attenuation coefficient of the specimen, which is proportional to the specimen’s mass density.

The application of absorption-contrast-based tomography to characterize a specimen inside a DAC allows one to measure the mass–pressure relation by measuring the volume change (volumetric approach) or, directly, the mass density change (mass density approach).

In high-pressure experiments with a DAC, the sample size is usually on the tens of micrometres scale. In the volumetric approach, a high-resolution microscope is therefore needed to measure the volume of such a sample precisely. As an example, if a sample has a size of 30 µm × 30 µm × 30 µm, the spatial resolution of the microscope has to be 0.3 µm to achieve 1% volume measurement precision. In practice, experimental data always suffer from various types of noise. This makes the determination of the volume boundary strongly dependent on the threshold. For two data sets taken under two pressures that have different noise levels, it is difficult to define consistent thresholds in two volume reconstructions. As a sequence, the volume determination from two volume reconstructions is subjected to subjective uncertainty. In the mass density approach, there is no need for a high-resolution microscope provided a homogeneous portion of the sample can be properly imaged. Most important is that the mass density approach can accommodate noise presented in the data. This is discussed below.

The filtered-back-projection (FBP) algorithm is widely used for tomography reconstruction (Kak & Slaney, 1987 ▶). With the FBP algorithm, the frequency spectrum of a sample’s projection images is filtered and then back-projected into the image space to obtain the sample structure (Natterer, 1986 ▶). When the projection images are noisy, the reconstructed sample structure suffers from errors. In this work, two types of noise that dominate the noise level in an image are considered, *i.e.* Gaussian-thermal noise related to the imaging detector’s dark current, and Poisson noise related to the detector’s shot noise. It can be proved that the expectation of FBP reconstructions from the noisy data of the sample is identical to that from the noise-free data of the sample (Kak & Slaney, 1987 ▶). Therefore, averaging reconstructions from multiple measurements of the same sample will reduce noise effects. In the numerical implementation of any algorithms, it is always subject to the error due to data discretization. To reduce the discretization error, averaging the reconstructed values in a small region, in which the mass density is supposed constant, will help. With the aid of two types of averaging the mass density with reduced noise effect can be obtained. The mass density of the sample obtained in this way is therefore a good approximation to its real mass density.

**Figure 1:**
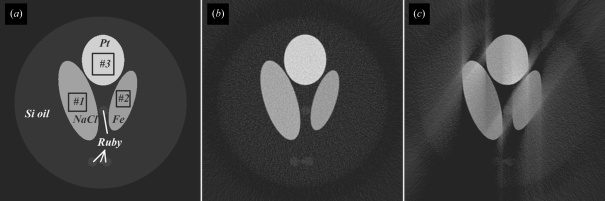
(*a*) Phantom used in the simulations. The pressure medium Si oil and pressure marker rubies were simulated. Three different samples, NaCl, Fe and Pt, which represent low-, middle- and high-density materials, were simulated. The boxes shown in the three samples are the regions in which the reconstructed mass densities are averaged. (*b*) Reconstruction from noisy complete data, and (*c*) reconstruction from noisy and incomplete data that had a missing angle of 35°. The display window is [−0.02, 2] in all three figures.

In high-pressure experiments with a DAC, the diamond cell is held by a steel frame. The sample is loaded between two diamonds; a gasket is generally used to form a closed chamber between the diamonds anvils and around the sample. To perform tomography of the sample in a DAC, the X-ray beam illuminating from the DAC side passes through the gasket onto the samples. The projection images obtained by rotating the DAC are recorded by an imaging detector downstream of the DAC. Owing to blocking by a steel frame, there are no projection images available in a certain angle range. With FBP, the reconstructions from tomographic data with missing angle usually suffer from distortions in terms of both reconstructed values and the shape of the sample, as illustrated in Fig. 1 ▶. In this case, however, the relative mass density of an unknown sample can still be obtained with some reference samples.

**Figure 2:**
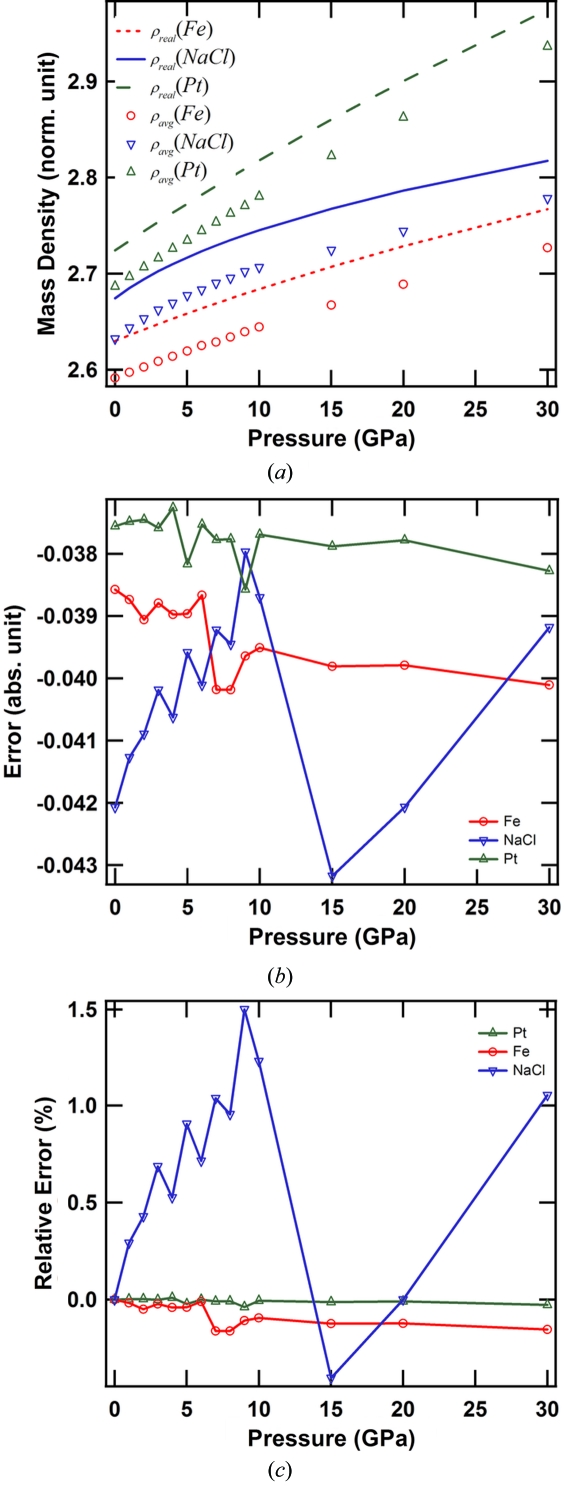
Reconstruction results with the complete data. (*a*) Ideal mass densities and calculated mass densities of NaCl, Fe and Pt. To show the details clearly, the curves associated with NaCl are offset by 2.4, and the curves associated with Fe are offset by 1.63. (*b*) Differences between the real mass densities and the tomographic estimations of the mass densities of NaCl, Fe and Pt. (*c*) Normalized error of the calculated mass densities in (*b*).

Simulations with the phantom shown in Fig. 1(*a*) ▶ have been carried out. In the phantom there are three different samples, NaCl, Fe and Pt, which represent low-density, middle-density and high-density materials. Pressure–mass-density (*P*–ρ) curves of NaCl, Fe (body-centred cubic) and Pt in the pressure range 0–30 GPa were calculated from their equations of state (Badro, 1999 ▶) and are shown in Fig. 2(*a*) ▶. Ruby balls as pressure markers and silicone oil as pressure medium are also simulated. The mass densities of NaCl, Fe and Pt under ambient condition are 2.16 g cm^−3^, 7.87 g cm^−3^ and 21.46 g cm^−3^, respectively. All materials’ mass densities were normalized to the mass density of Fe under ambient conditions. For simplicity, the mass densities of the ruby and the pressure medium were kept constant in the simulated pressure range, *i.e.* 2.73 g cm^−3^ and 1.06 g cm^−3^, respectively. No phase transitions of the samples were considered. The areas of three sample regions shrank accordingly, along with their mass density changes. A parallel illumination beam is assumed in the simulation. The width of the projection images was 512 pixels, and a total of 1024 projection images were evenly generated in the range 0–180°. The pixel size of the image is assumed to be 1 µm, which is typical in synchrotron-based microtomography. Poisson noise and Gaussian noise (having a mean level of 100 and deviation of 5) were added in the projection images. The illumination beam is uniform and has a pixel count of 3600. FBP with a Hann filter was employed to reconstruct the mass density distribution of the phantom from the simulated projection images. There were in total ten noisy data sets generated at each pressure point. Fig. 1(*b*) ▶ shows one of the ten reconstructions at 0 GPa.

First, the reconstructions from the complete tomographic data have been carried out. Equation (1) defines the error term used to characterize the errors in the reconstructions,
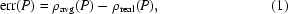
where ρ_avg_ is the mass density calculated from the tomographic reconstructions after a two-step average, and ρ_real_ is the mass density of the sample. Over the entire pressure range, as shown in Fig. 2(*a*) ▶, the calculated mass density curves of all three samples are visibly parallel to their ρ_real_ curves. Fig. 2(*b* ▶) shows the error curves calculated using equation (1). It is found that all error curves are close to zero and the magnitudes are small compared with ρ_real_ of samples. The offsets of the error curves from zero are due to the filtering used in the reconstructions, the residual error introduced by noise, and the residual discretization error. Since the magnitudes of the error curves are small compared with the samples’ mass densities over the entire pressure range, the following approximation can be made,
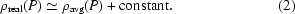
Because of the unknown constant in (2), the *P*–ρ curves of the samples calculated using (2) are in general offset from their real *P*–ρ curves. However, if a calculated *P*–ρ curve can be aligned to its corresponding real *P*–ρ at one pressure point, for instance at 0 GPa, the calculated *P*–ρ can be aligned to its real *P*–ρ curve. Fig. 2(*c*) ▶ shows the normalized error curves of three samples to their mass densities under ambient conditions. The error curves are aligned to the ambient conditions. As shown in Fig. 2(*c*) ▶, the error is within 2% for the low-density material (NaCl), and within 0.2% for the mid-density (Fe) and high-density materials (Pt). These results suggest that (2) is a good approximation of *P*–ρ measurement when there is no missing angle in the tomographic data. Heavier samples allow more precise results. It is interesting that the overall shape of the NaCl error curve is sinusoidal-like while that of the Fe and Pt error curves become more negative with decreasing pressure. This is probably related to the slopes of the *P*–ρ curves of the materials. As seen in Fig. 2(*a*) ▶, the slope of the NaCl error curve is large in the low-pressure range (<5 GPa), and is smaller and roughly constant in the higher-pressure range (15–30 GPa). The slopes of the Fe and Pt error curves are roughly constant in the range 0–30 GPa. The proof of this hypothesis is out of scope of this report.

**Figure 3:**
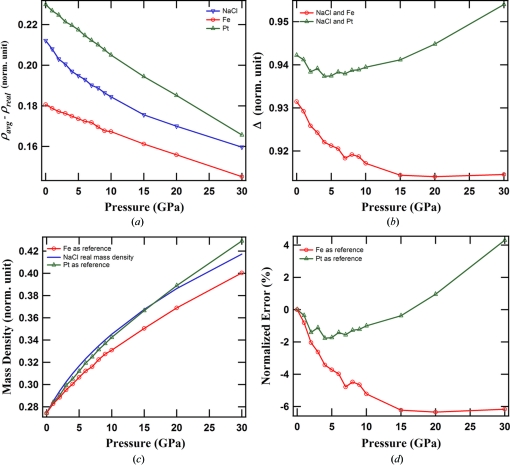
Results with the incomplete data. (*a*) Reconstruction errors of NaCl, Fe and Pt. Fe and Pt curves are offset by 0.69 and 0.96, respectively, to display the details of all three curves in the same figure. (*b*) The reconstruction error differences Δ between NaCl and Fe, and between NaCl and Pt. To show the details of both curves, the curves of the error difference between NaCl and Fe are offset by 0.21. (*c*) Calculated mass densities of NaCl using equation (3). The calculated curves are aligned to the real mass density curve at 0 GPa. (*d*) Normalized errors of the calculated mass densities in (*c*).

In the case of tomographic data with missing angle, the error curves calculated using equation (1) are no longer small in magnitude and widely separated from each other over the entire pressure range. In the simulation of this case, the same phantom was used and ten noisy data sets with 35° missing angle were generated. The 35° missing angle is typical of the Panoramic DAC cell that we used in the experiments. The error curves of three samples calculated using equation (2) are presented in Fig. 3(*a*) ▶. Fig. 3(*b*) ▶ shows the differences between error curves of NaCl and Fe, and NaCl and Pt. The fluctuation of the error curve associated with NaCl and Fe is small in the pressure range 0–15 GPa, and the fluctuation of the error curve associated with NaCl and Pt is small in the pressure range 15–30 GPa. Assume NaCl is the sample to be measured. If the fluctuation of the error curve between NaCl and a reference sample is small in some pressure range, the mass density of NaCl can be estimated in that pressure range using equation (3),
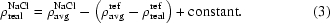
Here, 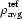 and 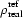 represent the calculated mass density of a reference sample from the tomographic reconstructions and the real mass density of the reference sample, respectively. Fig. 3(*c*) ▶ shows the calculated *P*–ρ curves with Fe and Pt as reference samples. The calculated curves are aligned to the real *P*–ρ curve of NaCl at 0 GPa. The normalized errors are shown in Fig. 3(*d*) ▶. It is seen that the calculated mass density of NaCl with Fe as a reference has good agreement (better than 0.2% in the range 15–30 GPa) with the real mass density curve on the higher-pressure end, while the calculated mass density of NaCl with Pt as a reference is roughly parallel to the real mass density curve on the lower-pressure end (within 2% in the range 0–15 GPa). The reference sample’s mass density curve, being more parallel to the mass density curve of the sample to be inspected, gives a better result. It should be pointed out that the normalized error of the relative mass density measurements is sample-dependent. The higher sample’s absolute mass density tends to have the smaller normalized error. For instance, if Pt is the unknown sample and NaCl is the reference in the pressure range 0–15 GPa, the normalized error will be about 0.1%. This is because the normalized error is determined by the difference between the error terms of the unknown sample and the reference sample, and the absolute mass density of the unknown sample. If the error term difference is the same, a higher estimated absolute mass density of the unknown sample makes the normalized error smaller.

The reason why the calculated mass density of NaCl with Pt as a reference has a good agreement with the real values in the pressure range 0–15 GPa is because the magnitude of (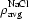 − 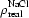) − (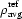 − 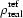) is small. This makes the approximation in equation (3) more pronounced. This also explains why the calculated mass density of NaCl with Fe as a reference has a relatively better agreement with the real curve within the pressure range 15–30 GPa. It is noted that, in Fig. 2(*b*) ▶, the *P*–ρ curve of NaCl is almost parallel to the *P*–ρ curve of Fe at the higher-pressure end, and to the *P*–ρ curve of Pt at the lower-pressure end. This is similar to the relations among the three error curves shown in Fig. 3(*c*) ▶. The similarity between the error curves and the mass density curves has a mathematical origin (see the Appendix *A* [App appa] for proof). Explicitly, the difference between the error terms of two samples is almost constant if the *P*–ρ curves of two samples are parallel to each other. This observation suggests that, for an unknown sample, a reference sample whose *P*–ρ curve has a similar slope to that of the unknown sample is desired. In practice, finding such a reference can be done iteratively. With an arbitrary reference sample, an estimated *P*–ρ curve of the unknown sample is obtained. If the estimated *P*–ρ curve is parallel to the reference sample’s *P*–ρ curve at the pressure of interest, the estimated *P*–ρ curve is accurate and a search is made. If two curves are not parallel, a new reference sample that has its *P*–ρ curve roughly parallel to the estimated curve of the unknown sample is chosen; this process is continued until the evaluation condition is satisfied. In choosing a reference sample, it is not necessary to have the candidate having its *P*–ρ curve parallel to the estimated *P*–ρ curve of the sample over a large pressure range. A large pressure range can be divided into several small ranges, and searching of the reference samples can be made in each small pressure range.

It is pointed out that the simulation model considered in this report is rather simple and idealized. There are many other factors that may affect the errors in a real experiment. One of these is the continuity of the sample. Loose powder samples that may have intergranular spaces filled with pressure medium are clearly not the case. The simulations in this report refer to samples in chunk state loaded into a DAC. For simplicity, Si oil and ruby compressions are not considered in the simulations. This, however, should not be a problem. As shown in the simulations, the materials (Pt and Fe) compressed under pressure do not significantly affect the *P*–ρ curve determination of the sample (NaCl). As discussed in Appendix *A* [App appa], the determination of the sample’s *P*–ρ curve is dominated by the mass density change of the sample. The effect of non-ideal geometry of sample chamber shape development during gasket material deformation upon compression is also neglected in the simulations. The effect of the gasket can be taken out by considering the absorption of the gasket of standard shape. In our experiments, hard X-rays and Be gasket are always used. The deviation of the gasket real shape from the assumed shape, and the part of the gasket deformation are small compared with the gasket size. The error from the non-ideal gasket shape can therefore be ignored. Other complicated issues such as phase mixture during phase transitions, crystallization and re-crystallization processes are neglected in the simulations. Errors contributed from inherent problems in high-pressure DAC experiments, such as pressure gradient, strain and stress states of sample embedded in non-ideal hydrostatic pressure medium, which are indeed very interesting topics and could be approached by other advanced novel techniques like synchrotron X-ray diffraction tomography, are out of the scope of this paper.

The proposed method has been applied to study the anomalous phase behaviour of amorphous Se (a-Se) (Liu *et al.*, 2008 ▶). In the experiments, silicone oil was used as the pressure medium and reference sample. Although, to the authors’ knowledge, there is no reported *P*–ρ result for silicone oil, our preliminary measurements of silicone oil’s *P*–ρ relation in separate experiments show that it is very close to the measured Se *P*–ρ relation with silicone oil as the reference sample. The reconstructed Se *P*–ρ curve shows that the mass density of a-Se at 10.4 GPa, where a-Se converts into monoclinic Se (m-Se) first and then m-Se converts into trigonal Se (t-Se), is in the middle of the mass densities of m-Se and t-Se. This suggests a density-fluctuation-driven phase transition from a-Se to m-Se.

## 3. Conclusions

In this work the methodology to measure mass density with tomography in high-pressure experiments is proposed. With numerical simulations, it is found that the relative mass density of an unknown sample can be obtained without reference samples in the case of complete data, and with reference samples when the tomographic data has missing angles. These simulation results support the validity of the proposed methods. In reality, there are many factors that may affect the mass density information extracted from tomography results. It is necessary to control the experimental conditions close to the condition employed in the simulations to make the methods valid.

## Figures and Tables

**Figure 4:**
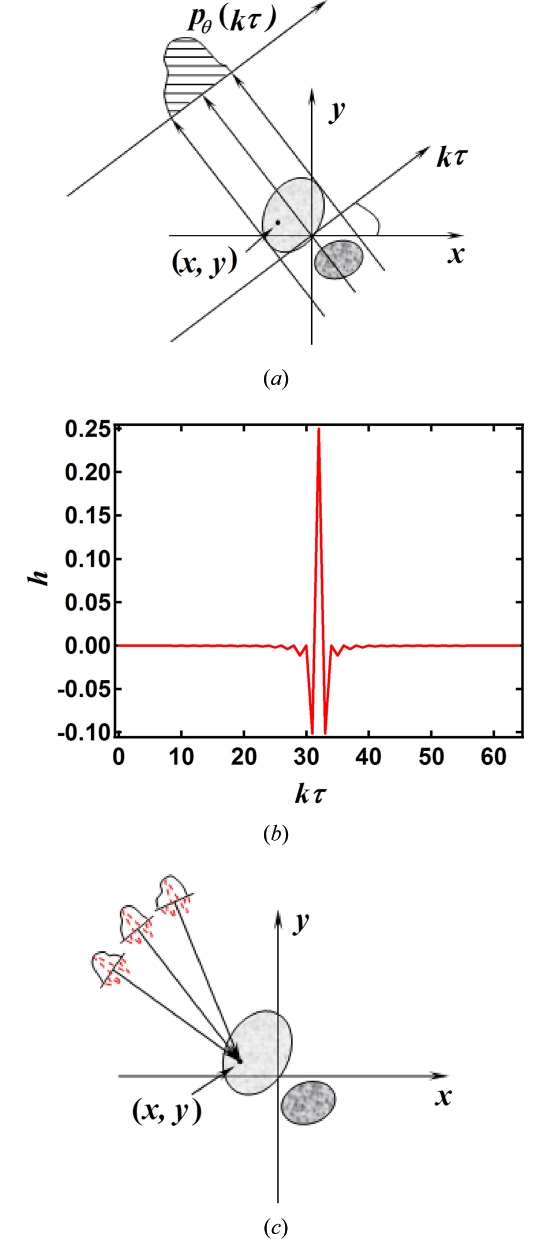
(*a*) Schematic of tomography. (*b*) Impulse response function used in the FBP reconstruction algorithm. (*c*) Schematic of FBP reconstruction. Back projecting the projections multiplied by the impulse response function gives the reconstruction at a specific position.

## Appendix A Heuristic argument for the correlation between mass density and its tomographic reconstruction error at a given point in an incomplete data case

In the parallel illumination beam case, a sample’s 3-D structure, with its linear attenuation coefficient as contrast, can be reconstructed using FBP (Kak & Slaney, 1987 ▶),

The coordinates definitions used in (4) are defined in Fig. 4(*a*) ▶. *p*_θ_ is the line integral of the sample’s linear attenuation coefficient along direction θ. The convolution kernel *h* is the tomography system’s impulse function, which is defined as

*h* is plotted in Fig. 4(*b*) ▶. As the linear attenuation coefficient is proportional to the sample’s mass density, (4) can be rewritten as

The prefactor *c* is the scaling factor between the sample’s mass density and the sample’s linear attenuation coefficient. The convolution of the projection *p*_θ_ with the impulse function *h* in (6) represents a filtering operation, and the summation over angle represents the backprojection operation. Fig. 4(*c*) ▶ schematically illustrates the FBP process represented in (6). Equation (6) can be rewritten as

Suppose the projection data are only being collected in the angle range  in an incomplete data case.  is the reconstructed mass density at position (*x*, *y*) from the data in the limited angle Ω, and δρ(*x*, *y*; *P*) is the reconstruction error.

In the so-called shift-and-add tomosynthesis, the reconstruction formula is exactly the same as the reconstruction formula for δρ(*x*, *y*; *P*) in (6) except for the different impulse response functions being used (Dobbins III & Godfrey, 2003 ▶). In tomosynthesis reconstructions, it can be proved qualitatively that the reconstructed value at one position is dominated by the mass density at that position, while a smooth background contributed from other positions is superimposed. This suggests that δρ(*x*, *y*; *P*) is also dominated by the mass density at the position (*x*, *y*). Therefore, the difference between δρ(*x*, *y*; *P*) at two positions reflects the mass density difference at two positions except for some background. If the *P*–ρ curves at two positions are parallel, the difference between δρ(*x*, *y*; *P*) at two positions is therefore constant. This confirms the observation in the numerical simulations.
